# The influence of interspecific interactions on species range expansion rates

**DOI:** 10.1111/j.1600-0587.2013.00574.x

**Published:** 2014-12-01

**Authors:** Jens-Christian Svenning, Dominique Gravel, Robert D. Holt, Frank M. Schurr, Wilfried Thuiller, Tamara Münkemüller, Katja H. Schiffers, Stefan Dullinger, Thomas C. Edwards, Thomas Hickler, Steven I. Higgins, Julia E. M. S. Nabel, Jörn Pagel, Signe Normand

**Affiliations:** (svenning@biology.au.dk), Ecoinformatics and Biodiversity, Dept of Bioscience, Aarhus Univ., Ny Munkegade 114, DK-8000 Aarhus C, Denmark; Dépt de biologie, chimie et géographie, Univ. du Québec à Rimouski, 300 Allée des Ursulines, Rimouski, QC G5L 3A1, Canada; Dept of Biology, Univ. of Florida, FL 32611, USA; Univ. Montpellier 2, CNRS, Inst. des Sciences de l’Évolution (UMR 5554), Place Eugène Bataillon, FR-34095 Montpellier cedex 05, France, and Inst. of Biochemistry and Biology, Univ. of Potsdam, Maulbeerallee 2, DE-14469 Potsdam, Germany; Evolution, Modeling and Analyzing of BIOdiversity group, Laboratoire d’Ecologie Alpine, UMR CNRS 5553, Univ. Joseph Fourier, Grenoble Cedex 9, France; Evolution, Modeling and Analyzing of BIOdiversity group, Laboratoire d’Ecologie Alpine, UMR CNRS 5553, Univ. Joseph Fourier, Grenoble Cedex 9, France; Evolution, Modeling and Analyzing of BIOdiversity group, Laboratoire d’Ecologie Alpine, UMR CNRS 5553, Univ. Joseph Fourier, Grenoble Cedex 9, France; Dept of Conservation Biology, Vegetation Ecology and Landscape Ecology, Univ. Wien, Rennweg 14, AT-1030 Vienna, Austria; USGS Utah Cooperative Fish and Wildlife Research Unit, Dept of Wildland Resources, 5230 Old Main Hill, Utah State Univ., Logan, UT 84322-5230, USA; Biodiversity and Climate Research Centre (BiK-F), Goethe-Univ. Frankfurt, Senckenberganlage 25, DE-60325 Frankfurt am Main, Germany; Botany Dept, Univ. of Otago, PO Box 56, Dunedin 9054, New Zealand; Landscape Dynamics, Swiss Federal Research Inst. WSL, Zürcherstr. 111, CH-8903 Birmensdorf, Switzerland, and Dept of Environmental System Science, Swiss Federal Inst. of Technology ETH, Universitätstrasse 16, CH-8092 Zurich, Switzerland; Univ. Montpellier 2, CNRS, Inst. des Sciences de l’Évolution (UMR 5554), Place Eugène Bataillon, FR-34095 Montpellier cedex 05, France, and Inst. of Biochemistry and Biology, Univ. of Potsdam, Maulbeerallee 2, DE-14469 Potsdam, Germany; Landscape Dynamics, Swiss Federal Research Inst. WSL, Zürcherstr. 111, CH-8903 Birmensdorf, Switzerland

## Abstract

Ongoing and predicted global change makes understanding and predicting species’ range shifts an urgent scientific priority. Here, we provide a synthetic perspective on the so far poorly understood effects of interspecific interactions on range expansion rates. We present theoretical foundations for how interspecific interactions may modulate range expansion rates, consider examples from empirical studies of biological invasions and natural range expansions as well as process-based simulations, and discuss how interspecific interactions can be more broadly represented in process-based, spatiotemporally explicit range forecasts. Theory tells us that interspecific interactions affect expansion rates via alteration of local population growth rates and spatial displacement rates, but also via effects on other demographic parameters. The best empirical evidence for interspecific effects on expansion rates comes from studies of biological invasions. Notably, invasion studies indicate that competitive dominance and release from specialized enemies can enhance expansion rates. Studies of natural range expansions especially point to the potential for competition from resident species to reduce expansion rates. Overall, it is clear that interspecific interactions may have important consequences for range dynamics, but also that their effects have received too little attention to robustly generalize on their importance. We then discuss how interspecific interactions effects can be more widely incorporated in dynamic modeling of range expansions. Importantly, models must describe spatiotemporal variation in both local population dynamics and dispersal. Finally, we derive the following guidelines for when it is particularly important to explicitly represent interspecific interactions in dynamic range expansion forecasts: if most interacting species show correlated spatial or temporal trends in their effects on the target species, if the number of interacting species is low, and if the abundance of one or more strongly interacting species is not closely linked to the abundance of the target species.

The pace and magnitude of global change makes understanding and predicting species’ range shifts an urgent scientific priority. Range expansion rates will constrain species distributions under rapid future climate change (Skov and Svenning 2004, Svenning and Sandel 2013), and understanding which factors govern those rates is critical for assessing the likely impacts of global change on biodiversity. The ability of species to track shifting environments not only influences their own survival, but also the dynamics of resident biotic communities in recipient communities (Ackerly 2003) as well as ecosystem processes (Svenning and Sandel 2013) and thus also services to people (Díaz et al. 2006).

Interspecific biotic interactions such as competition, predation, parasitism, facilitation, mutualism, and ecosystem engineering – as well as the rich array of indirect interactions emerging in networks – are often neglected in analyses of species ranges (Soberón 2007) and their dynamics. Recent theoretical and empirical studies highlight, however, that interspecific interactions may sometimes affect even species’ large-scale distributions by complementing or modulating the effects of abiotic conditions (reviewed by Case et al. 2005, Linder et al. 2012, Wisz et al. 2013). However, the general cross-scale importance of interspecific interactions for species distributions is still rather poorly understood.

Interspecific interactions not only affect equilibrial geographic distributions, but also range dynamics, by acting either as inhibitors, slowing down expansion rates, or as facilitators, speeding them up. Interspecific interactions may even cause range dynamics to halt unexpectedly, for instance via the existence of alternative stable states due to competitive priority effects (Belyea and Lancaster 1999, Urban and De Meester 2009) or via Allee effects (Keitt et al. 2001). Several recent reviews address how to incorporate interspecific interactions into species distribution models (Kissling et al. 2012, Linder et al. 2012, Wisz et al. 2013), and the impact of interspecific interactions on range dynamics has begun to receive attention in theoretical, empirical, and forecasting studies (Higgins et al. 2008, Triviño et al. 2011, Meier et al. 2012, Schweiger et al. 2012, Urban et al. 2012).

Here, we provide a synthetic perspective on the effects of interspecific interactions on range expansion rates and set the stage for their more general representation in forecasting models of range expansion. We first present a basic theoretical foundation for how interspecific interactions may modulate range expansions. We then review examples from empirical work on invasive species and natural range expansions as well as in process-based range simulations. We close with a synthesis as well as an overview of the consequences of interspecific interactions for studies forecasting range expansions, considering how biotic effects can be more widely incorporated, as well as providing guidelines for when this would be most needed.

## Basic theoretical framework

A rich and growing literature on mathematical models characterizing the spatial spread of single species has developed over the last several decades (Okubo and Levin 2001, Neubert and Parker 2004, Hastings et al. 2005, Caswell et al. 2011). All such models basically integrate a model for local demography with a model for movement. In principle, these spread models provide implicit first-order expectations about how interspecific interactions could influence range expansion rates, because the demographic and dispersal parameters in these models can be expressed as functions of the abundances, activity levels, or traits of interacting species, such as prey, predators, or competitors. So far there has not been much work on the impact of interspecific interactions specifically on expansion rates, particularly in complex multi-species communities (but see e.g. Fagan et al. 2005, Higgins et al. 2008, Gilman et al. 2010, Meier et al. 2012), although considerable work has been done in related areas (e.g. how biotic interactions may select for long-distance dispersal, Mueller-Landau et al. 2003). However, models of equilibrial range limits that explicitly include species interactions (Case et al. 2005) provide a natural starting-point for addressing this question, as they focus on fundamental demographic processes in each species, and on how those processes are coupled among species and play out across space.

Classical models of invasion (Fisher 1937, Skellam 1951, Okubo and Levin 2001) use a reaction–diffusion formulation in which local, density-dependent growth with continuously overlapping generations is represented by the per capita growth rate *f*(*N*), where *f* is assumed to decline with *N* (e.g. logistic growth), and the rate of random movement in a homogeneous environment is scaled by a diffusion coefficient, *D* (hereafter referred to as the spatial displacement rate). This classical reaction–diffusion model (Fisher–Skellam model) has the form

(1) $$\frac{\partial N}{\partial t}=f(N)+D\frac{\partial^{2}N}{\partial x^{2}}$$

**Figure 1:**
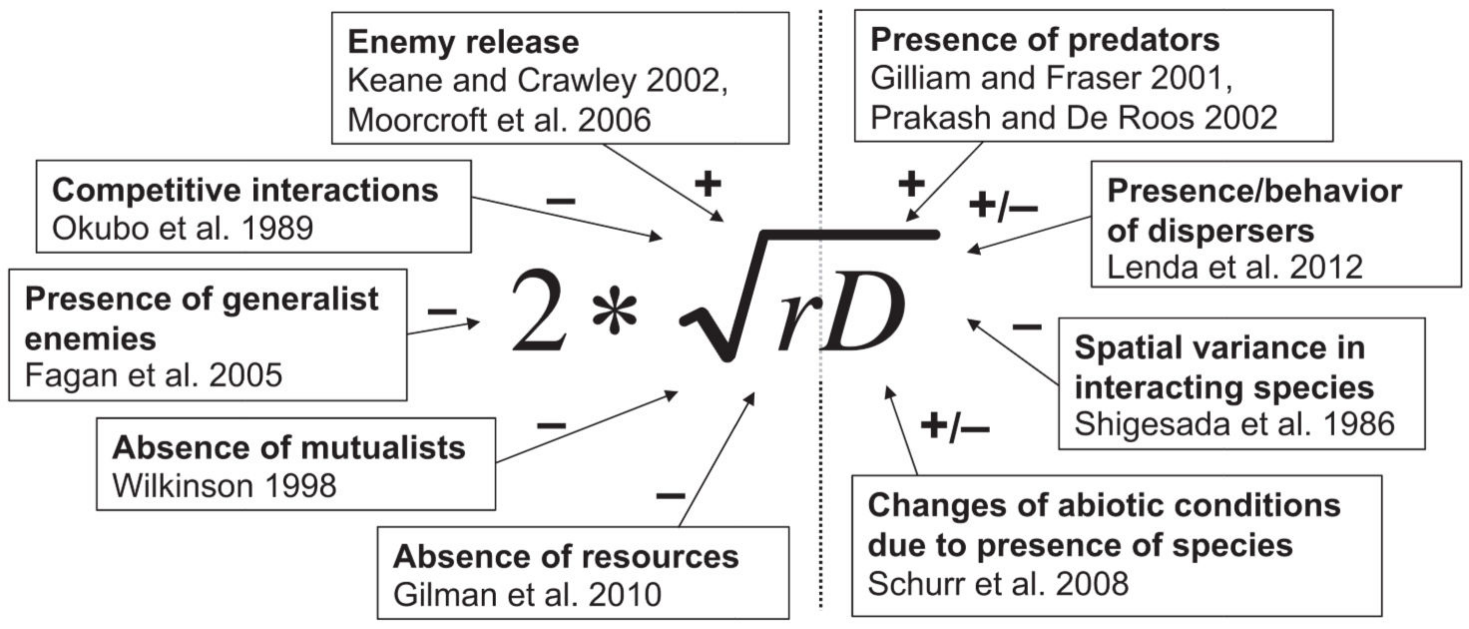
The equation for the asymptotic rate of spread of a population according to the classical Fisher–Skellam model and how its two parameters, *r*, the population growth rate (intrinsic rate of population growth at low densities) and *D*, the spatial displacement rate (the diffusion coefficient) may be affected by interspecific interactions, with a number of case studies highlighted.

The Fisher–Skellam model predicts that the asymptotic rate of spread of a population is:

(2) $$2\sqrt{rD}$$

where *r* = *f*(0) is the intrinsic rate of population growth at low densities. A key qualitative prediction of the Fisher–Skellam model is that expansion rate increases with both *r* and *D*. These quantities are not simply species traits, but more broadly reflect how a species interacts with its environment – including effects of other species, and indeed the entire network of interactions among species (Fig. 1). To describe such interactions, one can express *r* for a given time and location as

(3) $$r=r_{0}(E)+I$$

where *r*_0_(*E*) is the value of *r* for a given abiotic environment *E* in the absence of specific interspecific interactions. *I* is the overall effect of those interspecific interactions on *r*, with

(4) $$I=\Sigma_{i=1}^{S}\;N_{i}b_{i}$$

where *S* is the number of interacting species, *b_i_* is the per-capita effect of species *i* on the target species (which itself can be a function of a vector of species’ densities and local environmental conditions) and *N_i_* is the abundance of species *i*. In general, all components in the above expression can vary across space and through time. Notably, a fundamental feature of community dynamics under range shifts is that at any given location, new communities will emerge from immigration, emigration and extinctions. We thus expect a considerable change in *I* because of the reshuffling of the abundance vector *N*. If these shifts in abundance are very gradual across space, then the above reaction–diffusion model should provide a reasonable approximation for the rate of expansion of the focal species. However, if abundances or species composition change rapidly across space, this simple representation may break down, since diffusion leads to a kind of weighted averaging over these spatially varying conditions (Shigesada and Kawasaki 1997).

Theoretical studies reveal that interspecific interactions can considerably influence expansion rates via their impact on intrinsic growth rates (Fig. 1). For instance, expansion rates should decrease with an increasing strength of competitive interactions, which in effect reduces *r* (Okubo et al. 1989), and should therefore also be sensitive to species coexistence mechanisms and niche overlap (Münkemüller et al. 2009). Expansion rates may also be affected by mutualism and predator – prey dynamics. Range shifting rates should decline if generalist predators are present, elevating mortality rates, or mutualists are absent. Alternatively, enemy release can considerably accelerate range expansions (Keane and Crawley 2002). When a specialist enemy is lagging behind, an emergent ‘transient Janzen–Connell’ effect temporarily makes the migrating species a superior competitor, spreading more rapidly than might be expected from native populations where it co-occurs with its enemy (Moorcroft et al. 2006). The rate of spread of a specialist consumer can obviously be prevented by a slow expansion in the resource on which that consumer depends (Gilman et al. 2010). The same could hold for obligate, specialist mutualists (Wilkinson 1998). Conversely, rapidly dispersing specialist enemies may at times constrain the spread of their prey or hosts by spilling over the leading edge of an invasion (Fagan et al. 2005). Generalist predators might in general migrate faster than specialist enemies because of a higher probability of finding alternative prey species in new habitats (Gravel et al. 2011). In studies of island colonization, for example, there is evidence that generalist consumers colonize more rapidly than specialists (Holt 2010, Gravel et al. 2011), and the same pattern is likely to arise as communities shift in continental settings. This then would lower growth rates, and hence invasion speed, of some of the original prey species of those generalists. The transient co-distribution of interacting species in the context of the spatially and temporally shifting structure of the network of ecological interactions is therefore crucial to consider when assessing the impact of interspecific interactions on expansion rates.

Interspecific interactions may also affect expansion rates via effects on *D* that can be expressed in a form analogous to Eq. 3 (Fig. 1). Passively dispersed organisms often have evolved specialized adaptations to utilize mobile species as vectors, leading to biotically mediated long-distance dispersal events (Nathan et al. 2008). Changes to the abundance of these dispersal vectors can thus strongly affect the range expansions of dependent species. Interspecific interactions can even alter dispersal distances by abiotic vectors: for instance, neighboring trees may alter winddriven long-distance dispersal in wind-dispersed trees (Schurr et al. 2008). For species that can actively disperse, interacting species might bias movement and dispersal either by attraction (mutualists, host or prey species) or by repulsion (competitors, predators, parasites). The mere presence of predators in a habitat patch can for instance provoke prey to flee, thus incidentally enhancing the rate of colonization of empty habitable patches (Gilliam and Fraser 2001, Prakash and de Roos 2002).

## Recent theoretical developments and current challenges

Recent theoretical developments have gone beyond the Fisher–Skellam reaction–diffusion model in a number of different ways, with implications for our understanding of interspecific effects on range expansion rates. One basic direction is to allow each of several species to simultaneously disperse and interact, with coupling both via local growth rates and dispersal. This leads to equations like the Fisher–Skellam model above, but expanded to include multiple species distributed and moving in space. There is a reasonable body of theoretical literature taking this approach (reviewed by Murray 2001, Cantrell and Cosner 2003), but much remains to be done. This is a mathematically challenging area, and surprises can happen. For instance, if two species are competing according to a Lotka–Volterra model, spliced into a reaction–diffusion framework, and there is a combination of advection and dispersal by one competitor towards better-quality habitat, coexistence may arise at high advection, but not low advection (Cantrell et al. 2007).

The Fisher–Skellam model assumes continuous, overlapping generations. When generations do not overlap, discretetime integrodifference equations can be used to characterize range expansion (Hastings et al. 2005). This formulation can more readily account for a wide range of assumptions about the shape of dispersal kernels, which could vary with community composition. For instance, if each of several dispersal agents for a focal species has its own Gaussian dispersal kernel, but with different mean squared displacement values, the aggregate dispersal kernel for that species is non-Gaussian, with a fat tail. This leads to a higher rate of spread (Kot et al. 1996).

One obvious limitation of classical reaction-diffusion formulations for spread is that the environment is assumed to be spatially homogeneous and continuous. This assumption is unlikely to apply to range shifts, given that distributions are often initially constrained by spatial gradients in temperature and other environmental variables, and dispersal generally will occur across complex, heterogeneous and often patchy landscapes. Some work based on the Fisher–Skellam model does address heterogeneity; for instance, the rate of invasion decreases with the spatial variance in *D*, but is less impacted by spatial variance in *r* (Shigesada et al. 1986). Interestingly, given square-wave spatial variation, the quantity one substitutes for *r* in Eq. 2 is the arithmetic mean, but the harmonic mean for *D* (Shigesada et al. 1986). Given that the harmonic mean is disproportionately influenced by low values, spatial variation for *D* is expected to have a particularly strong impact on rates of spread. Spatial variation across communities in the abundance of interacting species – in particular dispersal vectors – could thus have systematic and strong effects on rates of range expansion. In patchy environments (where some areas simply cannot sustain a viable population at all), carrying capacity (*K*) and not just *r* can influence spread rates (Pachepsky and Levine 2011), for instance by permitting species at range margins to surmount threshold Allee densities (Keitt et al. 2001). Generalist predators can have switching responses, ignoring rare prey (hence having little or no impact on *r*) but inflicting mortality at higher densities, thus reducing the effective local carrying capacity and consequently lowering rates of invasion across patchy environments. With a fat-tailed dispersal kernel, it is more likely that individuals in populations near a range margin come from sites further in the interior of the range, where populations may be nearer carrying capacity. The higher that carrying capacity, the more dispersers will be available for colonization at the range margin.

Most species show temporal variation in their abundance, which means that the growth rate of any species with which they interact will also vary through time. Such temporal variation can alter expected rates of range expansion. Neubert et al. (2000) for example use a discrete generation integrodifference model to show that fluctuations in the abundance of a prey species can substantially slow the range expansion of a specialist consumer of that prey. Temporal variation in dispersal has been little studied, but is likely to be experienced by any species which depends upon other species for dispersal, since the abundances of abundance vectors will fluctuate over time. Ellner and Schreiber (2012) show that in some circumstances, temporal variation in dispersal can greatly speed up invasions.

The classical Fisher–Skellam model highlights the importance of intrinsic growth rates *r* in expanding populations, which provides a link between invasions and niche theory (Holt 2009). However, this class of models assumes that per capita growth rate is maximized at low *N*. Some species are likely to experience positive density dependence at low densities, i.e. Allee effects. In general, Allee effects slow down the rate of invasion (Taylor and Hastings 2005). Allee effects can emerge from interspecific interactions, for instance due to generalist predators that can be satiated. The emergent Allee effect in their prey is weakened if predator numbers are for any reason reduced. Mutualisms involve an indirect emergent Allee effect – an increase in mutualist A boosts the abundance or activity of mutualist B, which then feeds back with a time-lag to enhance the growth rate of mutualist A. This time-lag can occur within a generation when mutualist partners differ greatly in generation length (e.g. forest trees which depend upon insect pollinators).

Pairwise interactions are a natural starting point for analyzing how interspecific interactions alter population spread, but it should be kept in mind that most species are embedded in a complex network of interactions affecting their fecundity, survival, and dispersal (Bascompte and Jordano 2007). There is no theory yet on the impact of such complex network structures on range dynamics, but we may expect more specialized species – such as those relying on species-specific pollinators for dispersal – to be more sensitive to changes in the assemblage of coexisting species than species relying on a wide diversity of interacting species with some redundancy in function (Gilman et al. 2010). Likewise, losses of specific interacting species may have stronger effects on range dynamics in species-poor than species-rich communities in which single interactions are less important (cf. Schleuning et al. 2012).

Another limitation in classical reaction–diffusion approaches is that they assume abundances are large enough to be viewed as continuous variables. This is a dubious assumption at range margins. It is mathematically challenging to deal with demographic stochasticity and other processes that arise at small numbers, and in general one must rely on insights gleaned from non-analytic approaches such as individual-based simulations (Travis et al. 2005, Singer et al. 2013; see the section on ‘Representation of interspecific interactions in process-based range simulations’).

There may be a substantial evolutionary dimension to range dynamics, including both adaptation to local conditions and evolution of dispersal (Schiffers et al. 2013). Interspecific interactions are important for both of these. Holt et al. (2011), for instance, illustrate how predation, by affecting spatial variation in abundance in a prey species, can alter the relative importance of gene flow and selection in that species and thus the evolutionary constraints on range expansion. Long-distance dispersal is particularly important in governing range expansion rates, and escape from specialized natural enemies can be a powerful selection agent favoring such long-distance dispersal (Mueller-Landau et al. 2003). Ongoing coevolution may affect interaction strengths themselves, with contingent changes in rates of range expansion (Perkins 2012). A species expanding in its range is likely to encounter different suites of species and thus different blends of selective pressures. If it is initially somewhat maladapted in these new communities, and evolution increases mean fitness and hence its intrinsic growth rate, as it becomes better adapted to coping for instance with new natural enemies, etc., there can be an acceleration in the rate of invasion (Holt et al. 2005). One of the real challenges in developing eco-evolutionary models for range dynamics is characterizing how dispersal and local population sizes indirectly modulate evolutionary responses via controls on the pool of genetic variation available for local selection (Barton 2001). Low rates of dispersal could have a larger impact on range expansion via the infusion of genetic variation than is captured in purely ecological models.

## Empirical examples from biological invasions

**Figure 2:**
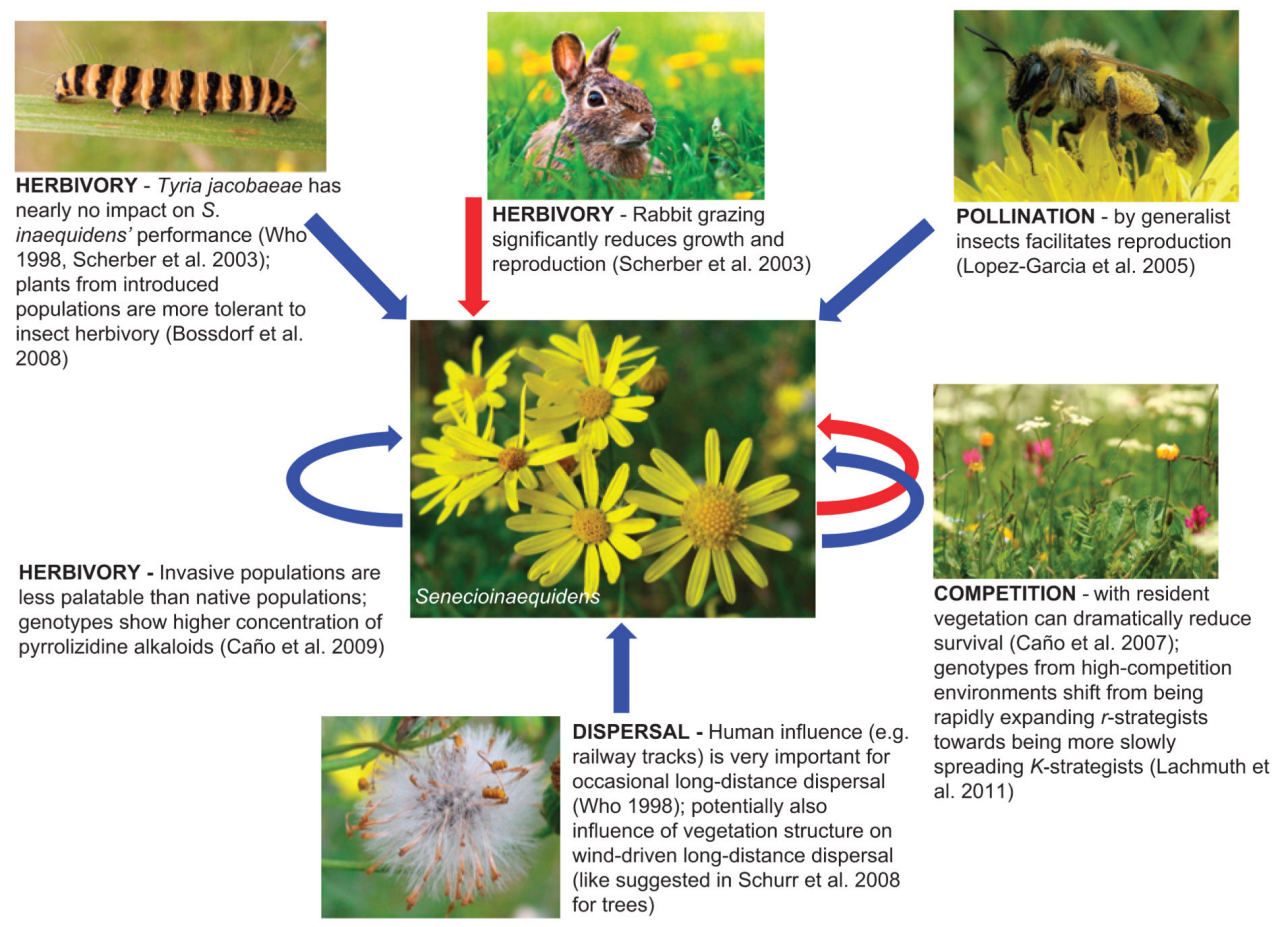
Overview of studied interspecific effects on the range expansion rate in the invasive plant *Senecio inaequidens*. Blue arrows indicate a positive effect on reproduction or dispersal, red arrows indicate a negative effect. Straight arrows indicate an external influence, curved arrows indicate an influence strongly mediated by the response of *Senecio inaequidens*. Sources: the photographs are by Kristian Peters (*Senecio inaequidens*, dispersal), Quartl (*Tyria jacobaeae*), OliBac (pollination), Benson Kua (rabbit), and Tamara Münke-müller (competition).

Perhaps the best empirical evidence for the influence of interspecific interactions on range expansion comes from the study of invading species (e.g. Fig. 2). Invasions are useful ‘natural experiments’ as one can study the spatiotemporal dynamics of one focal species, the invasive alien species (IAS), in the absence of long-term historical complexities. Furthermore, as they are often recent or ongoing phenomena and of importance for human activities such as conservation management or agriculture, there is often good data on IAS expansion rates.

Interspecific interactions, and more specifically competitive strength, predation, and mutualism, can influence IAS expansion rates via their impact on population growth rates (Fig. 2). This idea links to the concept of biotic resistance, whereby resident species one way or another repel invading species (Von Holle and Simberloff 2005). If competition is important one would expect IAS with strong niche differentiation compared to most native communities to be able to spread more quickly than those that either need to outcompete closely related species or co-occur with these through neutral dynamics. Even though this predicted effect on expansion rates has not been tested yet, several studies suggest that establishment can be fostered at the local scale when the niche of an IAS differs from the niches of the resident native species (Carboni et al. 2013). Other studies, however, have reported negligible importance of biotic resistance for invasion success relative to e.g. propagule pressure (Von Holle and Simberloff 2005). In addition to niche differentiation, the competitive abilities of plant invaders might also be increased by chemical weapons that are more successful in the introduced range because resident communities lack co-evolutionary adaptations (the ‘novel weapon hypothesis’, Callaway and Ridenour 2004). Moreover, *r* and *K* of IAS can evolve within the invaded range: this is, for example, the case in European populations of the South African ragwort *Senecio inaequidens* where genotypes from high-competition environments are less sensitive to competition, but invest less in reproduction, thereby shifting from being rapidly expanding *r*-strategists towards being more slowly spreading *K*-strategists (Lachmuth et al. 2011; Fig. 2).

Predation can have a strong influence on expansion rates as well and may interact with competition. In a common garden experiment with Chinese tallow *Triadica sebifera*, herbivores suppressed plant growth of invasive populations more than of native populations, especially for herbivore specialists, and when competition between neighboring plants was strong (Huang et al. 2012). There are a number of further examples which strongly suggest that the absence of specialized enemies can assist invasions either directly (Mitchell and Power 2003) or indirectly, e.g. via refugemediated apparent competition as in the case of the red alga *Bonnemaisonia hamifera* invading into the Atlantic (Enge et al. 2013). In general, release from specialist predators, consumers or pathogens, which confers competitive advantages on introduced species over native ones, is one of the most commonly accepted drivers of IAS dominance and spread in introduced ranges (Thuiller et al. 2010), even if the experimental evidence for the enemy release hypothesis is mixed (Keane and Crawley 2002). Positive interactions might also be involved in IAS spread. Many plants, for example, depend on pollinators for seed production. Although invasive plants usually seem well served by generalist pollinators and pollinator limitation is hence rarely a constraint to their spread (Richardson et al. 2000), the co-introduction of IAS plants and pollinators has been demonstrated to promote plant fecundity and spread rates (Stokes et al. 2006). The presence or absence of other mutualists might also heavily affect invasion rates. Exotic pines (*Pinus*) and other northern conifers are now widespread as invasive trees in the Southern Hemisphere (Richardson and Rejmánek 2004), but invasions are still limited in certain regions because adequate ectomycorrhizal fungi are lacking (Nuñez et al. 2009, Dickie et al. 2010). Similarly, growth of exotic legumes in Mediterranean coastal dune systems is depressed by the absence of co-evolved rhizobia from their native ranges (Rodríguez-Echeverría et al. 2012).

Interspecific interactions are also essential to the dispersal of IAS (Fig. 2). Bird and other vertebrate dispersal services to sessile organisms like plants are mutualistic interactions of particular relevance for range expansion rates. Although empirical studies are scarce, it is commonly thought that, similar to pollination, the dispersal pathways of IAS plants are rarely so specialized that disperser limitation could be a barrier to invasive spread (Richardson et al. 2000). Indeed, many fleshy fruited invasive plants are obviously non-selectively consumed and dispersed by generalist frugivorous birds (Gosper et al. 2005). A recent study on the invasion of North America by Oriental bittersweet *Celastrus orbiculatus* has, however, shown that dispersal by invasive alien European starlings *Sturnus vulgaris* has contributed considerably to its rapid spread by transporting large numbers of seeds over relatively long distances and depositing them at favorable micro-sites (Merow et al. 2011). Likewise, an invasive strangler fig *Ficus microcarpa* in Florida is more successful in establishing in landscapes that sustain higher abundances of fig-eating birds (Caughlin et al. 2012). In general, the effects of dispersal mutualisms on invasion success and alien spread rates seems an understudied issue, given its likely importance.

## Empirical examples from natural range shifts

Empirical evidence for impacts of interspecific interactions on expansion rates during natural range shifts is scarce and mostly only suggestive. This said, there are a number of studies that provide indications for important effects of interspecific interactions on expansion rates via either effects on population growth rates or spatial displacement (dispersal) rates. These studies have rather limited organismic scope, as they primarily concern trees, probably because trees are particularly well-studied with respect to past range shifts.

With respect to natural range expansions the main inter-specific interaction that has been considered is interspecific competition. In a number of cases of postglacial range expansions in trees, geographic variation in expansion rates has been linked to competition effects on population growth rates (*r*). For example, competition with other tree species has been mentioned as a possible explanation for cases where late arrival was associated with relatively slow subsequent local population expansions in certain tree species in the Alps (van der Knaap et al. 2005). Another example is the relatively delayed and spatially variable postglacial expansion of *Pinus banksiana* in Canada relative to *Picea* spp., which may be explained by its poor competitive ability and dependence on fire disturbance (McLeod and MacDonald 1997). Also suggestive of a biotic effect on expansion rates, human disturbance of forest stands have been shown to promote the spread or at least local expansion of *Fagus sylvatica* in parts of Europe, probably by reducing competition with other species (Küster 1997, Bradshaw et al. 2010). Indeed, human disturbance has been proposed as the factor that has allowed the much more northerly range expansion achieved by *Fagus sylvatica* in this interglacial relative to the previous Late and Middle Pleistocene interglacials (Lang 1994). Meier et al. (2012) provides a modeling example of such competitive effects on expansions, finding that they reduce expansion rates of late-successional much more than those of pioneer species.

**Figure 3:**
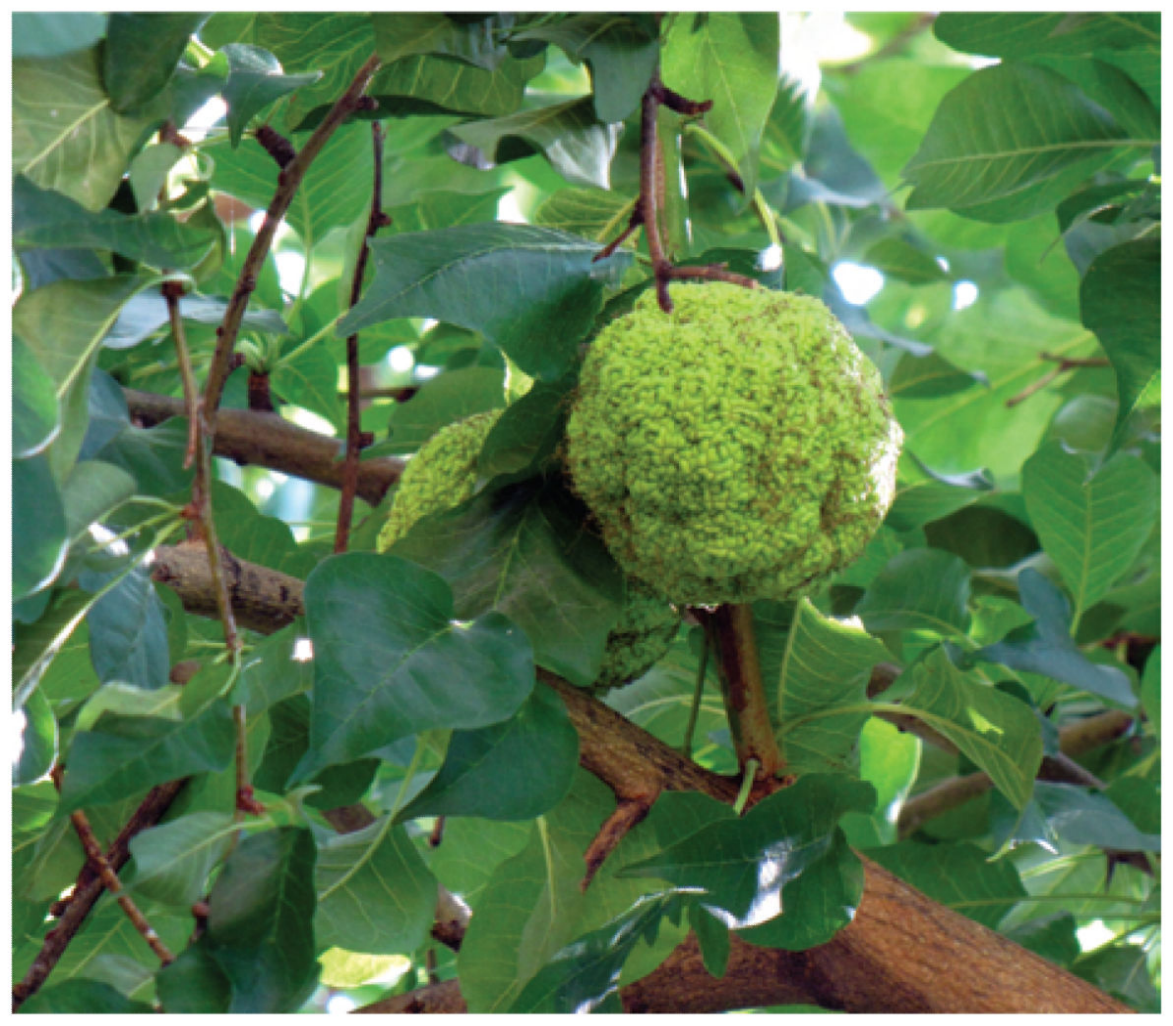
An intriguing, putative example of how interspecific interactions may affect range expansion rates in native plant species: the megafaunal losses at the end of the Pleistocene may have caused slow or failed range expansions in various endozoochorously dispersed trees (Janzen and Martin 1982), the osage orange *Maclura pomifera* with its large 8–15 cm diameter fruits being a prominent example (photo: J.-C. Svenning).

There are also empirical cases suggesting interspecific effects on expansion rates via effects on spatial displacement rates (*D*) by either affecting propagule or disperser dispersal distances or their mortality en route. A number of plant studies document how differences in disperser species or their behavior affect seed dispersal distances. During the last decades the southern European common walnut *Juglans regia* has begun a rapid expansion into natural vegetation in central Europe by seed dispersal from planted trees; this expansion is facilitated by climatic warming (Loacker et al. 2007), but also by an increasing abundance and changed behavior in its main disperser, the rook *Corvus frugileus* (Lenda et al. 2012). The walnut case also illustrates how human-mediated long-distance dispersal may strongly enhance range expansions within biogeographic regions (where it may not be reasonable to consider the expanders as invasive species), potentially enhancing the scope for range shifts in response to future warming (Skov and Svenning 2004, Van der Veken et al. 2008). Illustrating the opposite case, it has been proposed that the end-Pleistocene megafaunal losses have caused failed range expansions or even range contractions in various endozoochorously dispersed trees, e.g. the North American temperate species *Maclura pomifera, Gymnocladus dioicus*, and *Gleditsia triacanthos* (Janzen and Martin 1982) (Fig. 3). Extant large-bodied frugivorus species often play similarly important roles for long-distance dispersal, with their losses causing reduced dispersal distances and/or rapid evolutionary changes in seed size, potentially leading to population and range declines (Campos-Arceiz and Blake 2011, Galetti et al. 2013).

Overall, the scarce if suggestive empirical evidence indicates that interspecific interactions may have strong effects on expansion rates in natural range expansions, but clearly more research is required on this topic. Notably, there is a need to test for and quantify the role played by interspecific interactions, to expand studies to a wider range of other organisms, and to a broader suite of range expansions, e.g. contemporary as well as paleoecological range shifts.

## Representation of interspecific interactions in process-based range simulations

Many range modeling studies assume that the influence of interspecific interactions on species’ distributions is either weak or well correlated with abiotic environmental variables and thus does not need to be modeled explicitly. Yet interspecific interactions are explicitly represented in a class of ecological models called dynamic vegetation models (DVMs). DVMs are process-based simulation models of vegetation dynamics that explicitly consider birth, death, growth and how these processes are mediated by competitive interactions (Snell et al. pers. comm.). Not all kinds of interspecific interactions are currently covered in DVMs. Often these models only include competition for a subset of the following factors: water, nutrients, light and space (Scheiter et al. 2013), and only rarely do DVMs account for other interspecific interactions such as herbivory (Scheiter and Higgins 2012, Pachzelt et al. 2013). Furthermore, relatively few DVMs include dispersal dynamics and those that do have only considered small geographic extents due to high computational demands (Scheller and Mladenoff 2008, Nabel et al. 2013). Hence, the effects of interspecific interactions on expansion rates have seldom been explicitly studied with DVMs. However, the few existing DVM studies that do consider effects of interspecific interactions on expansion rates are suggestive. For example, in a simulation study with the forest landscape model LANDIS-II, Scheller and Mladenoff (2008) found that interspecific light competition reduced spreading rates. A study with the forest-landscape model TreeMig (Lischke et al. 2006) exploring how climate, competition and successional stages interact revealed that expansion rates are lower in established forests than in early succession forests, lower when more competing species are present and even lower when these competing species are late-successional species (Meier et al. 2012). This latter study’s qualitative findings are supported by empirical paleoecological studies (van der Knaap et al. 2005). The study of Meier et al. (2012) is also valuable since it illustrates at least one approach for overcoming the computational demands of DVMs that include dispersal, by integrating DVM-derived migration rates into a statistical species distribution model.

Spatial population models such as metapopulation models have also been extended to represent multiple species and their interactions. For example, a diffusion model of interacting populations successfully explains how competition with the native red squirrel *Sciurus vulgaris* slows the invasion of the grey squirrel *S. carolinensis* in Britain (Okubo et al. 1989). Moreover, a simulation model describing the local dynamics and dispersal of infected and uninfected foxes can explain the wavelike spread of rabies in Europe (Jeltsch et al. 1997). Such multispecies extensions of spatial population models thus have considerable potential for predicting range expansions of invasive or native species in a climate change context (Hastings et al. 2005, Kissling et al. 2012, Thuiller et al. 2013). Yet, although multispecies extensions have existed for a long time, they are still rarely applied to specific species and communities, but have mostly been used for more generalized analyses of how interactions affect spread (Travis et al. 2005, Singer et al. 2013). Related types of models such as approaches that link models of environmental tolerances with spatial population dynamics (e.g. the BioMove model of Midgley et al. (2010) or the dynamic range models discussed by Schurr et al. (2012)) also represent spatiotemporal dynamics, but do not yet explicitly consider biotic interactions.

## Synthesis

While interspecific effects on range expansion rates have received little attention so far, and especially so in empirical and fore- and hindcasting studies, it is clear that such effects may sometimes have important consequences for range dynamics. Theoretically, these effects may most obviously affect expansion rates via effects on both local population growth rates (*r*) and spatial displacement rates (*D*), but may also act via effects on other key quantities, such as carrying capacity. The strongest empirical evidence for the influence of interspecific interactions on expansion rates comes from studies of biological invasions. Notably, invasion studies indicate that competitive dominance and release from specialized enemies may enhance expansion rates. Although mutualisms may often be too generalized to limit expansion rates, in some cases, such as the northern conifer invasions in parts of the Southern Hemisphere (Nuñez et al. 2009), constraints on mutualisms clearly do limit expansion. Empirical evidence for interspecific interactions effects on expansion rates during natural range shifts is much weaker, but in a number of cases, such as postglacial range expansions in trees, competition is hypothesized to result in reduced expansion rates. Presence or abundance of dispersers is also hypothesized to enhance expansion rates in some cases. Process-based range simulations likewise indicate that competition may reduce expansion rates. Overall, there is emerging evidence that strong competitors may act as inhibitors on range expansion rates; interestingly, such species may themselves also have their own expansion rates particularly reduced by competition (van der Knaap et al. 2005, Meier et al. 2012). If there are tradeoffs among species between competitive ability and colonization potential, then these species may also differentially lag when communities respond to directional environmental change. Expansion rates can also be depressed by more diffuse competition, generalist natural enemies, and an overall low abundance of mutualists. While this review has shown that interspecific interactions may influence range expansion rates, it is also apparent that the empirical evidence is still too scarce to robustly generalize on their importance in absolute terms or relative to abiotic and intraspecific drivers.

## Consequences for forecasting range expansions

Models used to dynamically forecast range expansions and thus range expansion rates must – in one form or another – describe spatiotemporal variation in both local population dynamics and dispersal. At least, they therefore have to include both *r* and *D* (Higgins et al. 2003b). While there are a few models that do this in a spatiotemporally explicit fashion, very few models additionally account for how expansion rates are modified by interspecific interactions (see above). The scarcity of models forecasting spatiotemporal effects of interspecific interactions on range expansions is not surprising given the challenge of parameterizing potentially complex models of interacting meta-communities from limited available data on spatiotemporal dynamics and interactions (Kissling et al. 2012, Thuiller et al. 2013), as well as the computational challenges in running such models. Strategies for addressing these challenges, however, exist and are starting to be pursued (Kissling et al. 2012). For example, dynamic range models (DRMs) could, in principle, be applied to a broader range of systems and interaction types, even if they, in terms of biotic interactions, currently only describe effects of intraspecific density on *r* (Pagel and Schurr 2012). In the future, DRMs could be integrated with new statistical methods that enable the statistical estimation of interspecific interaction coefficients (Schurr et al. 2012) or with multi-species extensions of single-species physiological models (Kearney and Porter 2009, Higgins et al. 2012). For plants, progress may also be made by including dispersal and range expansion in a wider range of DVMs (Neilson et al. 2005, Snell et al. pers. comm.).

Although there is thus potential for progress, most forecasting models still take an ‘abiotic shortcut’ by linking *r* and *D* (or proxies thereof) to abiotic variables for which projections under environmental change exist. These models thereby implicitly, albeit incompletely so, account for variation in interspecific interactions that can be explained by these abiotic variables. Importantly, this abiotic shortcut may in some cases yield reliable spatiotemporal forecasts even if range expansions are strongly influenced by interspecific interactions simply because abiotic variables may be a good proxy of overall interaction effects (*I*) on *r* and *D*. For example, abiotic conditions can be a good proxy for vegetation biomass and net primary productivity and therefore potentially reflect large-scale variation in plantmediated competitive effects. This, however, assumes that these vegetation characteristics are not themselves subject to disequilibrium dynamics relative to the abiotic conditions (Svenning and Sandel 2013).

**Figure 4:**
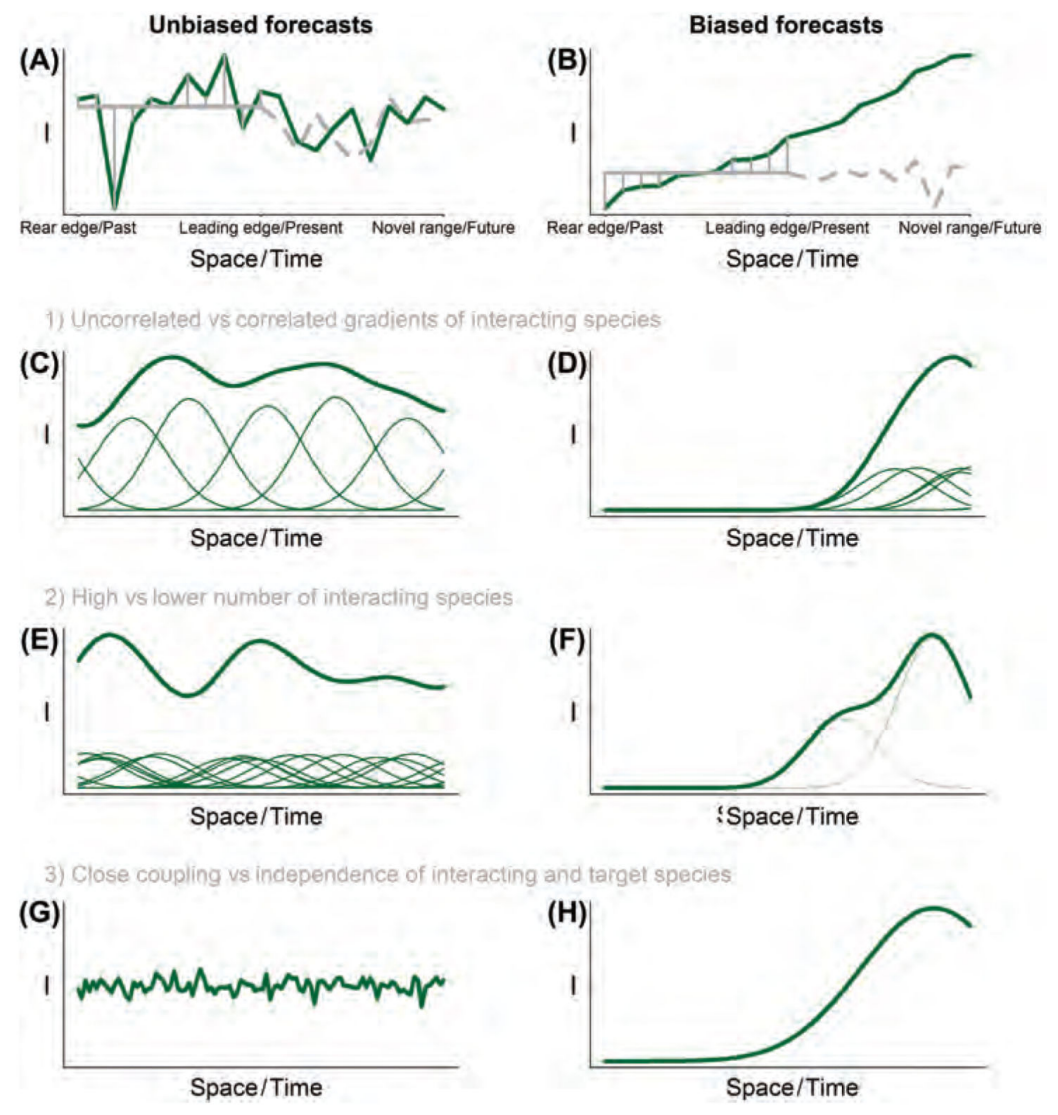
When are forecasts of species distribution models without explicit representation of interspecific interactions unbiased (left column) and biased (right column), respectively? (A) In general, forecasts will be unbiased if the summed effect of all interacting species (*I*, green line) shows no strong spatial or temporal trend. In this case, the effects of interspecific interactions can be reasonably approximated as stochastic deviations (vertical grey lines) from a single-species model (solid grey line). The hashed grey line shows a stochastic forecast of this single-species model. (B) In contrast, biased forecasts result if *I* shows a clear spatial or temporal trend. (C–H) These general considerations lead to three expectations for when the effects of individual interacting species (*N_i_b_i_*, thin lines) cause strong spatial or temporal gradients of *I* (bold lines) and will thus bias range expansion forecasts. Biased forecasts are less likely 1) if the effects of interacting species show uncorrelated (C) rather than correlated gradients (D), 2) if the number of interacting species is high (E) rather than low (F), or 3) if strongly interacting species are closely coupled with the target species (G) rather than being independent of it (H).

Moreover, forecasts may still be reliable even if the model does not describe variation in *I* (Fig. 4A), as forecast models typically contain stochastic terms that implicitly subsume the effects of interspecific interactions and other processes that are not resolved explicitly (Schurr et al. 2012). An abiotic shortcut model therefore interprets observed effects of variation in interspecific interactions as random deviations that follow a probability distribution (Fig. 4A, B). Forecasts extrapolate these deviations in space and time as uncorrelated draws from this distribution (Fig. 4A, B). This will not bias forecasts as long as the effect of interspecific interactions is well approximated by independent and identically distributed stochastic variables. In this case, as interspecific interactions become more important, the variance of the stochastic terms increases and the distribution of predicted range expansion rates simply becomes broader (i.e. more uncertain). However, this uncertainty about range expansion need not translate into uncertainty about the future fate of species as long as the distribution of predicted expansion rates lies mostly above or mostly below the velocity of environmental change (Higgins et al. 2003a).

A much more problematic situation arises if ignoring interspecific interactions causes upward or downward bias in forecasts of range expansion rates. Bias ensues if the effect of interspecific interactions on spatiotemporal variation in *r* and *D* is poorly approximated by independent and identically distributed random variables. In the following, we build on the above theoretical considerations to identify cases where ignoring interspecific interactions is most likely to bias range expansion forecasts and cases where the abiotic shortcut is expected to yield reliable forecasts. The bias caused by not explicitly representing *I* in forecasts depends on the magnitude and spatiotemporal variation of *I*. Obviously, the bias will be small if *I* is generally small. In the case of partially large *I*, range expansion forecasts will be strongly biased 1) if *I* changes systematically in space from the range core to beyond the leading edge, and/or 2) if *I* changes systematically in time from the beginning of data collection through the present to the future (Fig. 4B).

From these general considerations, we derive three expectations for when range expansion forecasts should explicitly represent interspecific interactions: 1) if most interacting species show similar spatial or temporal gradients of *N_i_b_i_*. In contrast to a situation of uncorrelated gradients (Fig. 4C), such correlated gradients (Fig. 4D) create directional changes in *I*. An example would be species expanding into new biomes where they interact with an entirely new set of species and may experience different interaction strengths. 2) If the number of interacting species (*S*) is low. *I* will tend to average out along the gradient if *S* is large (Fig. 4E), whereas it is more likely to vary systematically in space or time for low *S* (Fig. 4F). In addition, there is a greater chance of functional equivalence with large *S*. 3) If the abundance of one or more strongly interacting species *N_i_* are not closely linked to the abundance of the target species. This will be the case if the population dynamics of the interacting species is independent of the target species (e.g. for a superior competitor that occurs only in some regions, Fig. 4H). In contrast, effects of a host-specific pathogen with equal prevalence throughout the host range might be well approximated by a single-species model for the host (Fig. 4G).

We note that these processes can be interlinked: for instance, interactions tend to become more specialized at low *S* (Schleuning et al. 2012), making it more important to account for interspecific interactions.

## Outlook

This review shows that both theory and empirical studies suggest that interspecific interactions can have strong effects on species’ range expansion rates and thus on spatiotemporal community and ecosystem dynamics under future climate change. This said, the empirical evidence is still too scarce to robustly generalize on their importance in absolute terms or relative to abiotic and intraspecific drivers. In fact, it may well be that the effects of interspecific interactions are sometimes overwhelmed by these other drivers (cf. Von Holle and Simberloff 2005). Interspecific effects have hitherto only rarely and to a limited extent been incorporated in dynamic spatiotemporally explicit range forecasts. As outlined above lacking representation of interspecific effects may – depending on the circumstances – lead to noisy or biased forecasts, or have little consequence. Recent developments in methods for process-based, spatiotemporally explicit range forecasts have clear scope for being expanded to encompass interspecific effects. It should be a key priority in the development of dynamic forecasting models to pursue this goal. This challenge extends not just to model development per se, but also to further improve the theoretical and empirical basis. Notably, there is a clear dearth of studies that provide strong inference on the role of interspecific effects in warming-induced and otherwise natural range expansions.
